# Editorial: Drug repurposing to fight resistant fungal species: Recent developments as novel therapeutic strategies

**DOI:** 10.3389/fcimb.2025.1633037

**Published:** 2025-06-11

**Authors:** Maryam Roudbary, Marta H. Branquinha, André L. S. Santos

**Affiliations:** ^1^ Sydney Infectious Diseases Institute, Faculty of Health and Medicine, University of Sydney, Sydney, NSW, Australia; ^2^ Westmead Hospital, NSW Health, Sydney, NSW, Australia; ^3^ Instituto de Microbiologia Paulo de Góes, Centro de Ciências da Saúde, Universidade Federal do Rio de Janeiro, Rio de Janeiro, Brazil; ^4^ Rede Micologia RJ – Fundação de Amparo à Pesquisa do Estado do Rio de Janeiro (FAPERJ), Rio de Janeiro, Brazil

**Keywords:** fungal infection, drug resistance, therapeutic option, repurposing, novel antifungal compounds

Fungal infections represent a growing global health threat, driven by their increasing incidence, high mortality rates, and limited therapeutic options. They disproportionately affect at-risk populations such as immunocompromised individuals, transplant recipients, and patients undergoing intensive medical treatments. The situation is further exacerbated by challenges in early diagnosis, delayed treatment, and the rise of antifungal resistance. Additionally, climate change has emerged as a contributing factor, expanding the geographic range and virulence of pathogenic fungi, thereby intensifying the global burden of these infections. Supporting these concerns, recent estimates indicate a global annual incidence of approximately 6.5 million cases of invasive fungal infections – recognized as the most severe and treatment-resistant forms of mycoses – which are responsible for nearly 3.8 million deaths worldwide each year (Vitiello et al., 2023; Denning, 2024). Despite the pressing demand for effective antifungal treatments, current therapeutic options are hampered by several significant challenges. These include a restricted variety of drug classes, increasing rates of resistance, concerns regarding toxicity, suboptimal pharmacokinetics, and complicated drug-drug interactions. These factors collectively hinder the development and effectiveness of antifungal therapies, necessitating innovative solutions and more comprehensive strategies to improve patient outcomes (Meis et al., 2016; Corrêa-Junior et al., 2025).

In response to these challenges, there is a pressing necessity to uncover efficient and novel antifungal agents and/or repurposing of existing drugs introducing new mechanisms of action and avoid cross-resistance with current treatments. Additionally, optimizing combination therapies to mitigate resistance development has been identified as a strategic priority (Mota Fernandes et al., 2021). Of particular concern is *Candida albicans, Candida auris, Cryptococcus neoformans* and *Aspergillus fumigatus*, which were designated World Health Organization (WHO) critical priority fungal pathogen list (FPPL) in 2022 (World Health Organization, 2022). In alignment with this global call to action, the researchers are advancing the development of novel antimicrobial agents with promising antifungal properties aims to (i) enhance the antifungal efficacy, (ii) evaluate the activity against drug-resistant fungal species, and (iii) investigate synergistic combinations with existing antifungal agents to reduce the emergence of resistance.

The editors Maryam Roudbary, Marta Branquinha and André Santos proposed the publication of this Research Topic, titled *“Drug repurposing to fight resistant fungal species: recent developments as novel therapeutic strategies”*, in *Frontiers in Cellular and Infection Microbiology*. This initiative aims to advance the ongoing legacy of research in Medical Mycology while fostering the discovery and development of innovative therapeutic strategies to overcome drug resistance posing a therapeutic failure in healthcare system. This Research Topic serves as a dedicated platform for the dissemination of cutting-edge research, critical analyses, and emerging perspectives on the urgent challenge of antifungal resistance. It features a carefully curated collection of nine contributions, comprising six original research articles, one comprehensive review, one opinion piece, and one corrigendum. Collectively, these works reflect the depth of current progress in the field. To provide readers with a clear and engaging overview, concise summaries of each article are presented below.

The first paper, authored by Hu et al., describes two cases of *Pythium insidiosum* infection – one ocular and one cutaneous – diagnosed through metagenomic next-generation sequencing (mNGS). Zoospore induction enabled successful drug susceptibility testing, revealing that conventional antifungals and common antimicrobials were ineffective. However, significant *in vitro* activity was observed with minocycline, tigecycline, linezolid, erythromycin, and azithromycin. Guided by these findings and supported by zoospore-based susceptibility profiling, a tailored therapeutic approach was implemented, leading to marked clinical improvement and eventual recovery in the cutaneous case. The study highlights the importance of early diagnosis, appropriate anti-microbial selection and surgical intervention in managing pythiosis.

The study by Itoh et al. demonstrates that minocycline, when combined with caspofungin, exerts a synergistic antifungal effect, representing a promising therapeutic strategy against *Candida* infections, including drug-resistant strains, and surpassing rapamycin as a clinical candidate. This combination operates through a dual mechanism: it potentiates antifungal activity by simultaneously disrupting fungal cell wall integrity and inhibiting protein synthesis, while also modulating host immune responses by suppressing key signaling pathways – most notably through TOR (Target of Rapamycin) inhibition by minocycline. These findings underscore the enhanced clinical potential of minocycline–caspofungin co-therapy as an effective and multifaceted approach to combat *Candida* infections.

In the published study by König et al. highlights the dual antifungal and antiviral potential of ProcCluster^®^ and procaine hydrochloride, which are prodrugs derived from the local anesthetic procaine, against *Aspergillus* species and influenza A virus. Both compounds inhibited fungal growth, including triazole-resistant *A. fumigatus*, and reduced viral replication in lung epithelial coinfection models. ProcCluster^®^ disrupted fungal calcium homeostasis, a mechanism reversed by addition of calcium chloride. Interestingly, combination the antiviral favipiravir and ProcCluster^®^ showed enhanced activity against influenza A virus. These findings suggest promising broad-spectrum activity for these compounds in managing influenza-associated pulmonary aspergillosis.

The comprehensive review paper presented by Li et al. explores recent advances in using Artificial Intelligence (AI) and Machine Learning aim to strengthen the predictive power and clinical relevance of AI-driven insights in combating drug resistance. It discusses key models, such as Support Vector Machines, Random Forests and Deep Learning, while addressing challenges like data limitations and model interpretability. Additionally, the article emphasizes AI’s pivotal role in analyzing genomic data to uncover resistance mechanisms and highlights future directions to enhance predictive accuracy and cross-species applicability through algorithm improvement and interdisciplinary collaborations among bioinformaticians, microbiologists, and clinicians.

The original study published by Pan et al. investigates the antifungal properties of baicalin against *Candida albicans*, highlighting its ability to damage the fungal cell wall by unmasking β-1,3-glucan and increasing chitin deposition. RNA sequencing revealed that baicalin alters the expression of genes involved in cell wall biogenesis. This structural disruption enhances immune recognition, leading to increased phagocytosis and cytokine production by macrophages. These findings indicate baicalin not only possesses intrinsic antifungal properties, but also enhances host immune responses to facilitate fungal clearance of *C. albicans*.

The study conducted by Li et al. demonstrates that combining amantadine hydrochloride with azole antifungals, particularly fluconazole, exhibits a synergistic effect against drug-resistant *Candida albicans* both *in vitro* and *in vivo.* This synergy was attributed to enhance fungal clearance by inhibiting drug efflux pumps, reducing early biofilm formation and suppressing extracellular phospholipase activity. These findings support the potential of repurposing FDA-approved drugs like amantadine hydrochloride to improve antifungal therapy.

The paper published by Shah et al. evaluated 469 secondary metabolites from traditional Ayurvedic plants for antifungal activity against *Candida auris*, focusing on inhibition of lanosterol 14α-demethylase, a key enzyme in ergosterol biosynthesis. Through molecular docking, ADMET profiling, and molecular dynamics simulations, two compounds – *trans*-*p*-coumaric acid and (r)-n-(1’-methoxycarbonyl-2’-phenylethyl)-4-hydroxybenzamide – were identified as promising candidates. These compounds represented stable binding, favorable pharmacokinetics, low toxicity and strong potential as antifungal agents, highlighting their suitability for further experimental validation as treatments for drug-resistant fungal infections.

The study of Vanhoffelen et al. explores the antifungal synergy between posaconazole and tacrolimus against *Aspergillus fumigatus*, including azole-resistant strains. Prompted by a clinical case, the authors demonstrated that the combination significantly enhanced antifungal efficacy *in vitro*, especially against biofilm formation, and reduced fungal burden *in vivo* in *Galleria mellonella* larval model. These findings suggest that posaconazole–tacrolimus co-therapy may improve patient outcome on tacrolimus, warranting further validation in mammalian models and clinical studies.

In conclusion, this Research Topic brings together a selection of high-quality contributions that significantly advance the field of Medical Mycology, with a particular focus on innovative and alternative therapeutic approaches for combating fungal infections. The editors sincerely hope that the research presented herein will inspire curiosity, foster critical thinking and encourage continued exploration among students, early-career scientists and seasoned researchers alike. With deep appreciation, the editors extend their heartfelt thanks to all contributing authors for their outstanding work and invaluable collaboration. Their contributions not only enrich the scientific discourse with novel insights and meaningful advancement but also play a crucial role in elevating the visibility and relevance of Mycology within the broader context of contemporary biomedical research. The editors also wish to express their deep gratitude to the dedicated reviewers. Their expertise, thoughtful evaluations, and unwavering commitment to scientific rigor were instrumental in upholding the high standards and integrity of this Research Topic. Their efforts have made a lasting impact on the advancement of knowledge in this vital and evolving field.

## References

[B1] Corrêa-Junior D. Frases S. de S. Araújo G. R. (2025). Drug interactions of antifungal agents: clinical relevance and implications. Curr. Trop. Med. Rep. 12, 3. doi: 10.1007/s40475-024-00336-w

[B2] Denning D. W. (2024). Global incidence and mortality of severe fungal disease. Lancet Infect. Dis. 24, e428–e438. doi: 10.1016/S1473-3099(23)00692-8 38224705

[B3] Meis J. F. Chowdhary A. Rhodes J. L. Fisher M. C. Verweij P. E. (2016). Clinical implications of globally emerging azole resistance in *Aspergillus fumigatus* . Philos. Trans. R. Soc London B Biol. Sci. 371, 20150460. doi: 10.1098/rstb.2015.0460 28080986 PMC5095539

[B4] Mota Fernandes C. Dasilva D. Haranahalli K. McCarthy J. B. Mallamo J. Ojima I.. (2021). The future of antifungal drug therapy: novel compounds and targets. Antimicr. Agents Chemother. 65, e010128. doi: 10.1128/AAC.01719-20 PMC7848987 33229427

[B5] Vitiello A. Ferrara F. Boccellino M. Ponzo A. Cimmino C. Comberiati E.. (2023). Antifungal drug resistance: an emergent health threat. Biomedicines 11, 1063. doi: 10.3390/biomedicines11041063 37189681 PMC10135621

[B6] World Health Organization (2022). WHO fungal priority pathogens list to guide research, development and public health action (Geneva, Switzerland: World Health Organization).

